# Supplementary material for: The epidemiology of extrapulmonary tuberculosis in China: A large-scale multi-center observational study
Source: PLoS One. 2020 Aug 21;15(8):e0237753. doi: 10.1371/journal.pone.0237753 (PMC7446809; doi:10.1371/journal.pone.0237753)
Supplement: Attachment — Submitted filename: Response to Reviewers.docx [file pone.0237753.s002.docx]

PONE-D-20-17556
The epidemiology of extrapulmonary tuberculosis in China: A large-scale multi-center observational study
PLOS ONE

Dear Academic Editor Hasnain Seyed Ehtesham,

On behalf of my co-authors, we thank you very much for giving us an opportunity to revise our manuscript. We have tried our best to revise our manuscript according to the comments. At the same time, the revised version has been uploaded.

We are pleased to answer the questions raised by the academic editor and reviewers and the manuscript has also been revised according to the comments.

Journal Requirements:

1. Please ensure that your manuscript meets PLOS ONE's style requirements, including those for file naming. The PLOS ONE style templates can be found at

https://journals.plos.org/plosone/s/file?id=wjVg/PLOSOne_formatting_sample_main_body.pdf and

<https://journals.plos.org/plosone/s/file?id=ba62/PLOSOne_formatting_sample_title_authors_affiliations.pdf>

Answer: Thank you very much. Our manuscript meets PLOS ONE's style requirements.

2. Thank you for indicating in the Methods section that your ethics board provided approval and that they waived the need for informed patient consent because the study was retrospective and used anonymized data. Please additionally add this information to the Ethics Statement.

Answer: The information has been added to the ethics statement.

3. PLOS requires an ORCID iD for the corresponding author in Editorial Manager on papers submitted after December 6th, 2016. Please ensure that you have an ORCID iD and that it is validated in Editorial Manager. To do this, go to ‘Update my Information’ (in the upper left-hand corner of the main menu), and click on the Fetch/Validate link next to the ORCID field. This will take you to the ORCID site and allow you to create a new iD or authenticate a pre-existing iD in Editorial Manager. Please see the following video for instructions on linking an ORCID iD to your Editorial Manager account: <https://www.youtube.com/watch?v=_xcclfuvtxQ>

Answer: I have an ORCID iD and that it is validated in Editorial Manager.

4. Your ethics statement must appear in the Methods section of your manuscript. If your ethics statement is written in any section besides the Methods, please move it to the Methods section and delete it from any other section. Please also ensure that your ethics statement is included in your manuscript, as the ethics section of your online submission will not be published alongside your manuscript.

Answer: The ethics statement has been added to the Methods of the manuscript.

Reviewer's Responses to Questions

Reviewer #1: The article is good as it presents a huge data collected from 21 hospitals in the country. In the limitations the authors have mentioned that they could not compare the demographic details as income, housing, smoking etc. They also mentioned that they could not procure much data about paediatric tuberculosis.

They have only mentioned number of tuberculosis cases and concurrent pulmonary tuberculosis cases.

However, the following points need to be mentioned:

1. BCG vaccination policy of China and the vaccination status of the patients.

Answer: BCG vaccination policy of China: newborns are given BCG vaccination. BCG vaccination status of our group of patients was not investigated.

2. Immune status of the patients with underlying illnesses.

Answer: Sorry, the immune status of this group of patients was not detected.

3. The number of cases with previous TB infection.

Answer: Regrettably, previous TB infection in these patients was not included in our investigation.

4. Rate of cure and mortality.

Answer: Rate of cure and mortality of this group of patients were not investigated in this survey.

The authors have presented huge amount of data but they haven't correlated the data with the above mentioned points and have not related to any demographic details, that is required to be done.

Reviewer #2: Comments:
However, following suggestive comments will also construct this article stronger and beneficial for the readers:

Abstract:
• Word limits excided as per Journal guidelines. Should be modified while considering word limit.

Answer: According to the guidance of the magazine, the number of words in the abstract meets the requirements.

• Abbreviations of PTB are missing.

Answer: It has been modified as required.

Introduction:
• It is advised to add few points with recent references regarding diagnostic modalities and difficulties in diagnosing EP specimens as it is missing form introduction section.

Answer: We have added the EPTB diagnostic difficulties in the introduction.

Method:
• Author must specify about bronchial tuberculosis (14.96%), because this may consider under PTB.

Answer: Tracheobronchial tuberculosis (TBTB) is a type of tuberculosis that occurs in the mucosa, submucosa, smooth muscle, cartilage, and membrane of the trachea and bronchial. Previously bronchial tuberculosis was classified as extrapulmonary tuberculosis in China.

Results:
• Risk of EPTB is related to the degree of exposure to the pathogen and host immune factors like HIV, diabetes, malignancy etc. It’s my opinion that if author have the related data then this could make article more encouraging to readers.

Answer: Thank you very much for your suggestion. But, we don't have data on the degree of exposure to the pathogen and host immune factors in our survey.

Discussion:
• Line no. 282: Mycobacterium tuberculosis, write in italic.

Answer: In 282 lines of the manuscript, Mycobacterium Tuberculosis has been italicized.

Thank you very much for your attention and consideration. I am looking forward to hearing from you.

Yours Sincerely,

Shenjie Tang

Beijing Chest Hospital, Capital Medical University, Beijing Tuberculosis and Thoracic Tumor Research Institute, Beijing, 101149, China

Email: [tangsj1106@hotmail.com](mailto:tangsj1106@hotmail.com), [tangsj1106@vip.sina.com](mailto:tangsj1106@vip.sina.com)
